# Insulin Resistance Is an Independent Determinate of ED in Young Adult Men

**DOI:** 10.1371/journal.pone.0083951

**Published:** 2013-12-31

**Authors:** Shengfu Chen, Rongpei Wu, Yanping Huang, Fufu Zheng, Yangbin Ou, Xiangan Tu, Yadong Zhang, Yong Gao, Xin Chen, Tao Zheng, Qiyun Yang, Zi Wan, Yuanyuan Zhang, Xiangzhou Sun, Guihua Liu, Chunhua Deng

**Affiliations:** 1 Department of Urology, First Affiliated Hospital of Sun Yat-Sen University, Guangzhou, Guangdong Province, China; 2 Department of Urology, Shanghai Institute of Andrology, Renji Hospital, Shanghai Jiaotong University School of Medicine, Shanghai, China; 3 Wake Forest University, Institute for Regenerative Medicine, Winston-Salem, North Carolina, United States of America; University of Michigan Medical School, United States of America

## Abstract

**Background:**

Insulin resistance (IR) triggers endothelial dysfunction, which contributes to erectile dysfunction (ED) and cardiovascular disease.

**Aim:**

To evaluate whether IR was related to ED in young adult patients.

**Methods:**

A total of 283 consecutive men complaining of ED at least six months were enrolled, with a full medical history, physical examination, and laboratory tests collected. Quantitative Insulin Sensitivity Check Index (QUICKI) was used to determine IR. The severity of ED was assessed by IIEF-5 questionnaire. Endothelial function was assessed by ultrasonographic examination of brachial artery flow mediated dilation (FMD).

**Results:**

IR was detected in 52% patients. Subjects with IR had significant higher total cholesterol, triglycerides, low density lipoprotein-cholesterol (LDL-c), glycated haemoglobin (HBA1c), high sensitivity C-reactive protein (hs-CRP) and body mass index (BMI), but showed significant lower IIEF-5 score, FMD%, high density lipoprotein -cholesterol (HDL-c), testosterone, sex hormone binding globulin (SHBG) levels than patients without IR. Multiple regression analysis showed QUICKI and testosterone were independent predictors of IIEF-5 score. Furthermore, the incidence of IR was correlated with the severity of ED.

**Conclusions:**

Compared with other CVFs, IR was found as the most prevalent in our subjects. Besides, IR was independently associated with ED and its severity, suggesting an adverse effect of insulin resistance on erectile function.

## Introduction

Erectile dysfunction (ED) is defined as the persistent inability to achieve and maintain a sufficient erection to complete intercourse [1]. The incidence of ED in patients less than 40 years old was 22.1%–35% [2], [3]. ED is now considered as a vascular disease [4]. The prevalence and severity of ED increases with number of cardiovascular risk factors (CVFs)[5]. CVFs start early, go through young age and manifest as cardiovascular disease (CVD) in middle-aged and/or elderly populations [6]. Therefore, young men with ED may provide a good opportunity for early CVFs assessment.

Insulin resistance (IR), defined as decreased sensitivity and/or responsiveness to metabolic actions of insulin that promote glucose disposal, is one of the important CVFs. IR may damage endothelial function which is characterized by decreased nitric oxide (NO) release and elevated levels of endothelin [7]. IR is a part of prediabetic state and the progression of IR to diabetes parallels the progression of endothelial dysfunction to atherosclerosis [7]. Gotoh et al found that IR significantly increased the risk for developing CVD [8]. In addition, IR predicted atherosclerosis plaque progression in both the diabetic and non-diabetic population [9].

ED shared the same risk factors, i.e. CVFs and common pathogenesis, i.e. endothelial dysfunction with CVD. Based on the “artery size hypothesis” [10], it is supposed that IR raises endothelial dysfunction, which could manifest as ED and earlier than CVD or other vascular complications. Moreover, young men with ED are more predictable to develop subsequent CVD than the elderly [11]. Therefore, studying the relationship between IR and ED in young men is of great importance for the prevention and treatment of ED and secondary CVD.

Glucose clamp is a “gold standard” to determine insulin sensitivity in vivo, but the method is time-consuming, labor intensive, and expensive [12]. Quantitative Insulin Sensitivity Check Index (QUICKI) has been reported as the most accurate surrogate index for determining insulin sensitivity [13]. The QUICKI depends on fasting insulin and fasting glucose levels, with lower levels representing greater degrees of IR.

We hereby aimed to assess the prevalence of IR in young ED patients and then to further analyze the relationships between IR and ED.

## Participants and Methods

### Study population

A total of 318 patients were recruited from the andrology outpatient population of our hospital from October 2011 to December 2012. The general information including lifestyle; anthropometrics, psychosocial attributes, medication and surgical history was collected via a validated questionnaire by fully-trained interviewers. All the patients underwent a complete physical examination and blood pressure measurement in the sitting position. Smokers were defined as individuals smoking at least one cigarette per day for more than one year. An individual with an average daily intake of more than 30 ml alcohol was considered as “drinker”. Subjects' height and weight were measured and BMI was calculated using the formula: BMI = weight (kg)/height^2^ (m^2^).

Inclusion criteria for all the patients included age between 18 to 45 years, a history of ED at least six month, a stable female sexual partner, IIEF-5 score <21, and absence of a questionable/clinical symptoms of cardiovascular disease or a history of cardiovascular disease.

Exclusion criteria were the patients having a possible symptoms of cardiovascular disease (chest tightness, chest pain etc.), or a history of cardiovascular disease including ischemic heart disease (angina or myocardial infarction), heart failure; cerebrovascular disease (transient ischemic attacks or strokes); peripheral vascular disease; testosterone deficiency; a history of drug abuse; psychiatric disease; pelvic, spine or brain trauma or surgery; overt endocrine, liver, lung or kidney disease; malignancies; hematological disorders; abnormal urogenital status, such as small testicle (<2 cm), and penile plaques. In addition, individuals experiencing self-identified major lifestyle changes (endurance exercise, weight reduction program) or significant changes in psychological status (loss of job, divorce) in the past 6 months were also excluded.

Written informed consents were obtained prior to data collection and the study was approved by the Ethics Committee of the First Affiliated Hospital of Sun Yat-Sen University, Guangzhou, China. The study was conducted in accordance with the Declaration of Helsinki to protect personal data. Each participant was assigned a number for keeping his privacy.

### Erectile function assessment

Erectile function was assessed based on a comprehensive medical and sexual history. The International Index of Erectile Function-5 (IIEF-5) was used for diagnosis and classification. The degree of ED was classified according to the IIEF-5 scores as severe (5–7), moderate (8–11), mild-moderate (12–16), mild (17–21), or none (22–25). In addition, penile duplex ultrasonography was used to evaluate the organic factors derived from anatomic abnormalities affecting penile erection.

### Laboratory tests

Venous blood samples were drawn from every individual between 8:00 to 10:00 AM after 12–14 h overnight fasting. After centrifugation (3,000 rpm at 25°C) for 10 minutes, the serum samples were stored at −80°C until analysis. Laboratory tests including fasting blood glucose, insulin, lipid profile, total testosterone, and hs-CRP were determined by using commercially available assay kits in the Clinical Testing Center of our hospital. The laboratory technicians were blinded to the clinical characteristics of the patients.

### Endothelial function assessment

The procedure was conducted between 8 to 10AM after patients being abstaining from alcohol, tobacco, coffee, vasoactive agents and food for 8–12 hours before the examination mainly according to previous description [14], [15]. Subjects rested in a supine position for about 15 min before examination. Briefly, the right brachial artery was evaluated using a high-resolution ultrasound machine equipped with a high-frequency and linear array ultrasound probe (5–12 MHz, VIVID7 GE, USA). The probe was positioned approximately 2–5 cm proximal to the elbow. Diameter and blood flow velocity of brachial artery were recorded by R-wave gating of the synchronic electrocardiogram (ECG). After baseline measurements, an arterial pressure cuff was placed around the arm inflated above 250 mmHg for 5 min before deflating. After cuff deflation, the greatest diameter was recorded at end diastole (tip of ECG R-wave). FMD was calculated as the percentage change in diameter before and after cuff inflation.

### Definition of terms

Hypertension was defined as systolic blood pressure ≥140 mmHg, diastolic blood pressure ≥90 mmHg. Impaired fasting glucose (hyperglycaemia) was defined as a fasting plasma glucose level in the range 5.6–7.0 mmol/L and diabetes was defined as fasting plasma glucose ≥7.0 mmol/L. IR was evaluated using the QUICKI method according to the formula: QUICKI  = 1/(log fasting insulin + log fasting glucose) and IR was defined as a QUICKI value ≤0.357 [16]. Hypercholesterolemia was defined as serum total cholesterol >6.22 mmol/L. Hypertriglyceridemia was defined as triglycerides ≥2.26 mmol/L. High LDL-cholesterol was defined as ≥4.14 mmol/L. Low HDL-cholesterol was defined as <1.04 mmol/L, and high lipoprotein-α was defined as >300 mg/L. Cutoffs for total cholesterol, triglycerides, HDL-c, LDL-c and lipoprotein-α were determined according to the Chinese Guidelines on Prevention and Treatment of Dyslipidemia in Adults [17]. The elevated serum free fatty acids (FFA) for fasting patients was defined as >0.6 mmol/L [18]. Hyperuricemia was defined as a serum uric acid level ≥420 ummol/L [19]. Subjects with hs-CRP levels >3 mg/L were considered as having a high hs-CRP concentration. Testosterone deficiency was defined as serum total testosterone level less than 250 ng/dL [20].

### Statistical analysis

The distribution of all the variables was firstly determined using the Kolmogorov–Smirnov test. And the data were presented as median (interquartile range) or as mean ± SD according to result of Kolmogorov–Smirnov test. Correlation between CVFs and IIEF-5 scores were analyzed by using the Pearson correlation or Spearman rank correlation analysis if the data did not meet the bivariate normally distributed variables. Patients were subdivided into two groups according to their IR status. ED patients without IR were defined as control group. Student's t test for normally distributed variables and Mann-Whitney U test for nonnormally distributed variables, as appropriate; Moreover, the analysis of contingency tables and the associated chi-square statistics were used. Stepwise multiple regressions were used to determine the significant independent contribution of CVFs to ED. A P-value<0.05 (two sides) was considered significant. Data analyses were performed by using SPSS 13.0 software.

## Results

In total, 318 ED patients were recruited in the study. Twenty-eight patients were excluded because of age >45 years. Five patients were excluded due to pelvic surgery or injury history, and two other patients were excluded because of mental illness. Finally, 283 men (mean age 32.9±6.8 years) were included for analysis.

**Figure 1:**
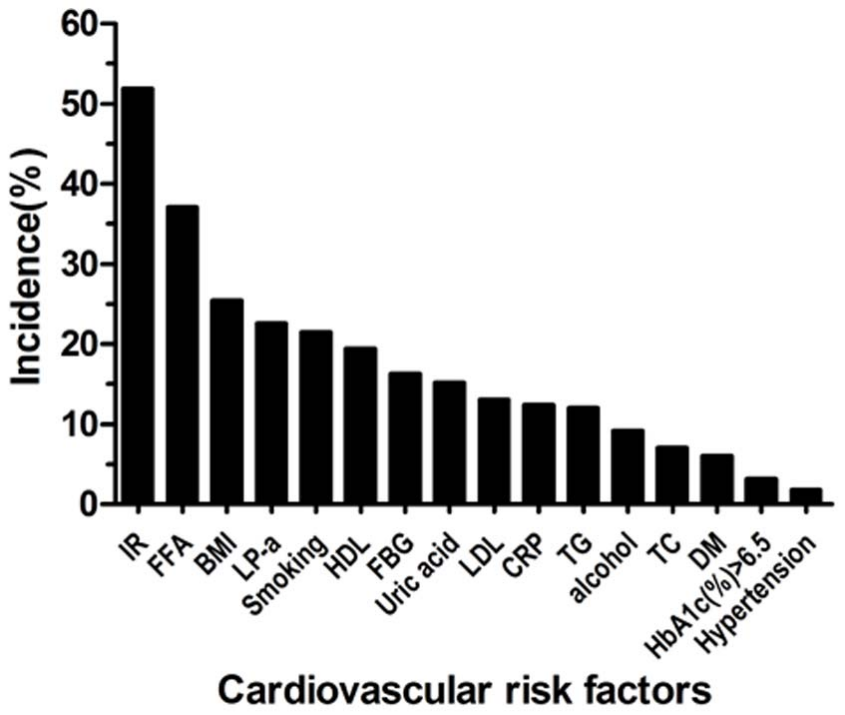
The incidence of various cardiovascular risk factors in 283 young ED patients. IR, insulin resistance; LDL-c, Low-density lipoprotein cholesterol; FFA, Free fatty acids; TC, Total Cholesterol; BMI, Body mass index; LP-a,Lipoprotein-a; TG, Triglycerides; HDL-c, High- density lipoprotein cholesterol; IFG, Impaired fasting glucose; hs-CRP, high sensitivity c-reactive protein; DM, Diabetes Mellitus; HbA1c(%), Glycosylated hemoglobin; Various risk factors overlap and therefore do not add to 100%.

The most prevalent potential risk factor was IR (52.0%) followed by increase in FFA (37.1%) and abnormal BMI (25.4%) in these patients. Prevalence of diabetes was 17 men (6%) including 9 men (3.2%) newly diagnosed diabetes. Only 5 men were diagnosed as having hypertension (1.8%) and three men suffered both hypertension and diabetes (**Figure1**).

Significant positive correlations were found between QUICKI and IIEF-5 scores (*p* = 0.005), FMD%(p = 0.014), total testosterone (*p*<0.001), SHBG (*p*<0.001), and HDL-c (*p*<0.001). Moreover, QUICKI significantly correlated inversely to BMI (*p*<0.001), triglycerides (*p*<0.001), LDL-c (*p*<0.001), total cholesterol (*p*<0.001), TG/HDL (*p*<0.001), LDL/HDL (*p*<0.001), HBA1c (*p*<0.001), uric acid (*p* = 0.04), and hs-CRP (*p*<0.001). However, there were no correlations between QUICKI and FFA, or lipoprotein-α (**Table 1**).

**Table 1:** Spearman correlations between QUICKI and other variables in young ED patients.

Variables	Correlation coefficient	P
Body mass index (kg/m^2^)	−0.439	<0.001
IIEF-5 score	0.165	0.005
Total Cholesterol (mmol/L)	−0.218	<0.001
Triglycerides (mmol/L)	−0.372	<0.001
LDL-c (mmol/L)	−0.214	<0.001
HDL-c (mmol/L)	0.263	<0.001
TG/HDL	−0.389	<0.001
LDL/HDL	−0.329	<0.001
Free fatty acids (umol/L)	−0.088	0.141
Lipoprotein-a (mg/L)	0.079	0.187
Uric acid(umol/L)	−0.122	0.040
Hs-CRP(mg/L)	−0.254	<0.001
Glycated serum protein (umol/L)	0.207	<0.001
HBAc1 (%)	−0.332	<0.001
Total testosterone (ng/dL)	0.293	<0.001
SHBG(nmol/L)	0.355	<0.001
FMD%	0.146	0.014

IIEF-5, 5-item International Index of Erectile Function

Questionnaire; LDL-c, Low-density lipoprotein cholesterol;

HDL-c, High- density lipoprotein cholesterol; hs-CRP, High

sensitivity c-reactive protein; HbA1c(%), Glycosylated hemoglobin;

SHBG, Sex hormone binding globulin; FMD, flow mediated dilation.

Subjects with IR were more obese than those without IR (*p*<0.001), and had higher levels of triglycerides (*p*<0.001), total cholesterol (*p* = 0.004), LDL-c (*p* = 0.011), hs-CRP (*p*<0.001) and HBAc1 (*p* = 0.001), but lower levels of total testosterone (*p*<0.001) SHBG (*p*<0.001), IIEF-5 scores (p<0.001) and FMD% values (p = 0.039) compared with controls (**Table 2** and **Figure S1**). There were no significant difference in FFA, lipoprotein-α and uric acid of the patients with or without IR.

**Table 2:** Clinical characteristics in men with ED associated or not associated to IR (QUICKI<0.357).

Variables	Total (n = 283)	QUICKI<0.357 (n = 147)	QUICKI≥0.357 (n = 136)	*P*
Age, years	32(28,38)	31(28,39)	32(27,38)	0.312
Body mass index, kg/m^2^	23.45±3.19	24.59±3.19	22.22±2.70	<0.001
Smoker(non/current)	222/61	113/34	109/27	0.503
IIEF-5 score	14(10,17)	13(9,17)	15(12,19)	<0.001
Insulin, IU/ml	6.7(4.8,10.0)	9.8(8.1,12.1)	4.7(3.8,5.9)	<0.001
Fasting glucose, mmol/L	5.2(4.8,5.6)	5.4(5.1,5.8)	4.9(4.7,5.3)	<0.001
GSP, umol/L	243(215,271)	231(210,262)	254(225,280)	0.001
HbA1c(%)	5.4(5.2,5.7)	5.6(5.3,5.8)	5.3(5.2,5.6)	0.001
Triglycerides, mmol/L	1.05(0.73,1.50)	1.21(0.89,1.96)	0.85(0.64,1.27)	<0.001
HDL-c, mmol/L	1.26(1.10,1.46)	1.19(1.02,1.39)	1.35(1.16,1.50)	<0.001
TG/HDL	0.82(0.53,1.38)	1.04(0.68,1.66)	0.61(0.46,1.06)	<0.001
LDL-c, mmol/L	3.19±0.83	3.31±0.87	3.06±0.76	0.011
LDL/HDL	2.54(1.91,3.17)	2.79(2.15,3.34)	2.18(1.77,2.91)	<0.001
Total cholesterol, mmol/L	4.88±0.91	5.03±0.95	4.72±0.84	0.004
Free fatty acids, umol/L	503(372,715)	495(400,733)	515(344,693)	0.481
Lipoprotein-a, mg/L	187(149,286)	181(145,268)	194(151,313)	0.320
Uric acid, umol/L	327(270,383)	331(270,393)	319(264,379)	0.201
hs-CRP, mg/L	0.68(0.29,1.58)	0.87(0.45,1.99)	0.48(0.25,1.14)	<0.001
Total testosterone, ng/dL	586(476,736)	541(432,666)	635(540,781)	<0.001
SHBG, nmol/L	34.0(25.1,44.1)	30.6(20.6,36.9)	38.1(30.8,50.0)	<0.001
FMD%	9.14±2.56	8.84±2.53	9.47±2.57	0.039

IIEF-5 score, 5-item International Index of Erectile Function questionnaire. GSP, Glycated serum protein;HbA1c (%), Glycosylated hemoglobin; HDL-c, High- density lipoprotein cholesterol; LDL-c, Low-density lipoprotein cholesterol; hs-CRP, High sensitivity c-reactive protein; SHBG, Sex hormone binding globulin; FMD, flow mediated dilation;

Comparison between patients with and without IR by unpaired t-test, Mann-Whitney U-test or Chi-square test depending on the data distribution. Data are expressed as mean ±SD or as median (interquartiles range) for skwed variables

P*, patients with QUICKI<0.357 compared with patients with QUICKI≥0.357.

**Figure 2:**
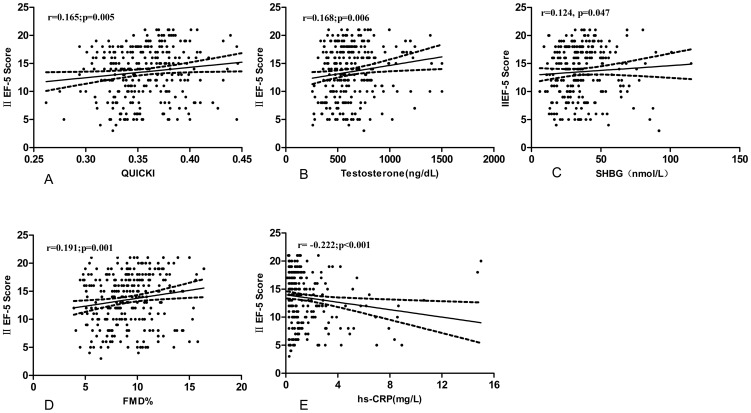
Spearman correlation of the IIEF-5 score with QUICKI, testosterone, SHBG, FMD% and hs-CRP in our subjects.

**Figure 3:**
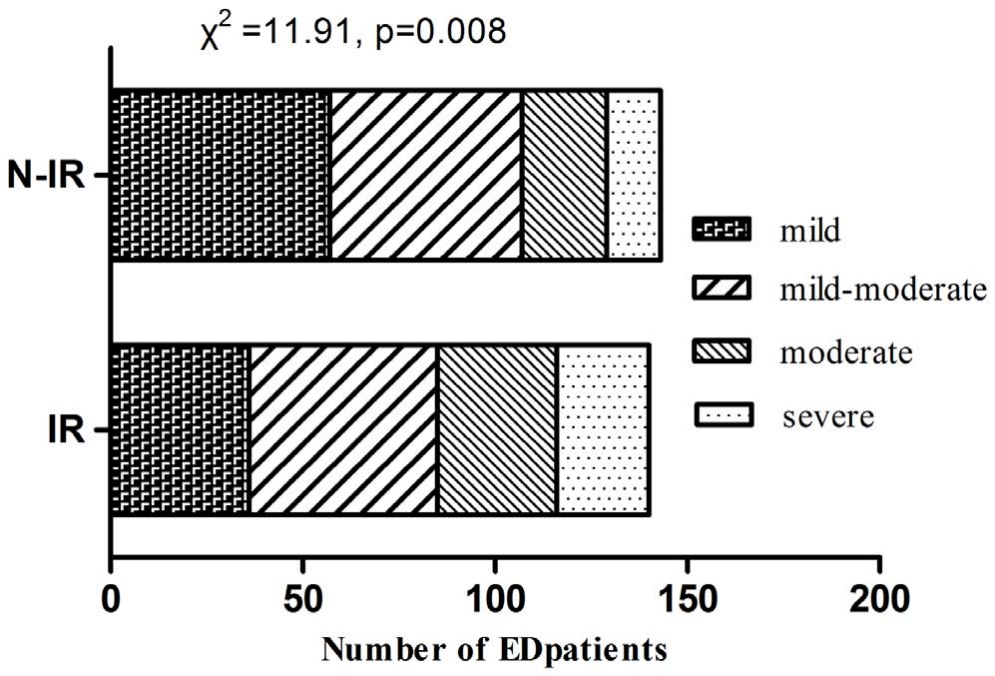
Patients with different degrees of erectile dysfunction in IR and N-IR state.

Apart from QUICKI, total testosterone, SHBG and FMD% were positively related to the IIEF-5 score, while hs-CRP showed negative relation with the IIEF-5 score (*p*<0.05, respectively) (**Figure 2**). QUICKI and testosterone were independent predictors of IIEF-5 score (*p*<0.05, respectively) according to the multiple stepwise regression analysis (**Table 3**). Furthermore, IR was associated with the severity of ED (χ^2^ = 11.91, *p* = 0.008) (**Figure 3**). IR was found in 26/38 (68.4%) of patients with severe ED, 33/53 (62.3%) of patients with moderate ED, 51/99 (51.5%) of patients with mild to moderate ED, 37/93 (39.8%) of patients with mild ED.

**Table 3:** Stepwise regression analysis of including varieties of CVFs as independent variables and IIEF-5score as a dependent variable.

	Unstandardized		Standardized					
Variable	Coefficients		Coefficients		t	sig	95%CI for B	
	B	Std.Error		Beta			Lower	upper
Testosterone	0.359	0.164		0.170	2.187	**0.030**	0.035	0.683
QUICKI	23.506	11.156		0.164	2.107	**0.037**	1.476	45.536

Dependent Variable: IIEF-5score.

## Discussion

This study found IR was common (52%) in the young ED patients without clinical cardiovascular disease. Moreover, the ED patients with IR had more CVFs and IR was independently associated with ED and its severity. Our results suggested an adverse effect of insulin resistance on erectile function and physicians should conduct a comprehensive assessment of undiagnosed risk factors besides psychological factors when they face young ED patients.

Since PDE5 inhibitors are effective in the majority of young ED patients, they are often prescribed to psychological problems, a population who may indeed suffer potential organic disorders. However, our previous study showed that some young ED patients who were traditionally considered as psychological ED actually possessed higher glycosylated serum protein levels compared to the non-ED patients [21]. Psychological stress may result in a deregulation of inflammatory and neuroendocine systems and an impairment of vascular function [22], [23]. In the present study, IR was detected in 52% young ED patients without clinical cardiovascular disease, which was slightly different from previous study. Bansal et al showed 79.2% of ED patients and 73.3% of nondiabetic population were defined as IR by using QUICKI [24]. And only 27% of general population had IR reported by another study [25]. Therefore, the incidence of IR is varied in the different target population. Although our target population was very young (32.9±6.8 years), IR was detected in over half of the ED patients indicating that IR is related to erectile dysfunction. In a case–control study, a higher degree of IR has been found in men with ED aged 40 to 70 years than that of aged-matched normal control [26]. According to our data, IR indeed inversely correlated with ED and the incidence of IR increased with the severity of ED. After adjustment for other factors including metabolic, biochemical and hormonal parameters, IR was still shown to be an independent contribution to ED and correlated with the severity of it, suggesting that IR may play a role in the development of ED.

However, the specific mechanisms of how IR results in ED remain unclear. The most possible explanation is the endothelial dysfunction [27], which is confirmed in the present study. IR may impair the PI3K-dependent signaling, alter the balance between NO and endothelin-1 towards diminished insulin-induced vasodilation or even vasoconstriction leading to exacerbated IR and endothelial dysfunction [28]. Conversely, enhanced insulin responses will increase production of NO by induced expression and activation of eNOS [29]. Besides, neural NO signaling is also impaired due to oxidative stress and neural NO synthase uncoupling in penile arteries under conditions of IR [30].

Another potential reason is the relatively low testosterone levels in men with IR. Testosterone secretion of Leyding cell decreases in the IR condition [31]. Androgens are necessary for erectile function and testosterone is important to the maintenance of peripheral nitric oxide synthesis. Physiological concentrations of testosterone can increase NO synthesis which may improve the endothelial NOS (eNOS) expression and phosphorylation by activation of intracellular signaling pathways and Ca^2+^ influx in vitro cultured human endothelial cells [32], [33]. Testosterone therapy has been found to restore diabetes-induced ED and sildenafil responsiveness in two diabetic animal models [34].

Furthermore, IR is also associated with other cardiovascular risk factors or disorders. According to our data, QUICKI was significantly related to BMI. Moreover, the BMI of young ED patients with IR was higher than those without IR. The results show that BMI is an important predictor of insulin resistance. Dyslipidemia, which characteristic as high plasma triglyceride concentration, reduced HDL-c concentration, and increased concentration of LDL-c, is one of the key risk factors for CVD in diabetes mellitus [35]. In our study, TG/HDL and LDL/HDL, which are considered as a better predictor of metabolic syndrome and insulin resistance than apolipoprotein B/apolipoprotein A1 [36], were negatively correlated with QUICKI, indicating the close relationship between dyslipidemia and IR. However, based on our results, this tendency was manifested as early as prediabetic stage. As dyslipidemia is an important risk of atherosclerosis, early intervention exerted at the prediabetic stage may reduce the ratio of complications.

Increased plasma FFA levels are considered as an important cause of obesity-associated IR and CVD [37]. Some studies showed that elevated FFA contributes to IR independently of obesity [38]. However, according to our data, there were no relationships between FFA levels and IR, or FFA levels and BMI. The possible reason is that fasting FFA represents only a short period of a whole day and there are other factors associated with IR [37].

In addition, insulin is thought to regulate testosterone secretion and inhibits SHBG synthesis and secretion. Obese and insulin-resistant men with higher insulin levels would be expected to have lower SHBG levels and consequently lower total testosterone concentrations [39]. Our results showed that testosterone and SHBG levels were significantly lower in patients with IR (QUICKI <0.357) than non-IR (QUICKI ≥0.357) controls and both were negatively correlated with the IR.

In the current study, men with IR were clustered with simultaneous increase of adverse factors including elevated cholesterol, LDL, triglycerides, hs-CRP, HBAc1 (%), overweight and decreased positive factors such as lower HDL, testosterone and SHBG levels. Most of these changes are related to endothelial dysfunction [40]. These unfavorable changes may play a synergistic role in further impairing endothelial function in IR condition, as confirmed by lower FMD in ED patients with IR. Taken together with previous reports, which have demonstrated that ED is a predictor of CVD [4], it is reasonable to conclude that young men with IR are prone to a high-risk of suffering ED and CVD.

Some limitations of this study must be acknowledged. Firstly, since this is a cross-sectional study, the causal relationship between IR and ED could not be determined. Secondly, some other CVFs such as family history of cardiovascular disease, physical activity and the possible effect of drugs to ED and IR were not evaluated. In addition, we could not fully discriminate organic from psychogenic ED, but all the cases had symptoms for at least 6 month and 52% of patients were found to have IR regardless of organic or psychogenic ED. Finally, we employed QUICKI in the study, whereas many other studies were based on HOMA-IR or fasting serum insulin as a marker for IR. Thus, it may be hard to compare our results with those of other studies because there are no standardized diagnostic criteria or methods to define IR currently. However, we believe that these limitations do not affect the interpretation of our results as a whole.

In conclusion, the present study showed that IR is independently associated with ED and its severity, indicating that IR may be a sign of ED and subsequent CVD. Besides, decreased endothelial function and testosterone level were seen in ED patients with IR, suggesting the possible mechanism of IR in the pathophysiology of ED. In clinical practice, early diagnosis and treatment of IR should be a part of the therapy plan for young adult men with ED.

## Supporting Information

Figure S1 **The comparison of IIEF-5 score, lipid profile, hs-CRP, total testosterone, FMD% between IR group and non-IR group.**
(TIF)
