# Supplementary figures and images for: Insulin Resistance Is an Independent Determinate of ED in Young Adult Men
Source: PLoS One. 2013 Dec 31;8(12):e83951. doi: 10.1371/journal.pone.0083951 (PMC3877124; doi:10.1371/journal.pone.0083951)

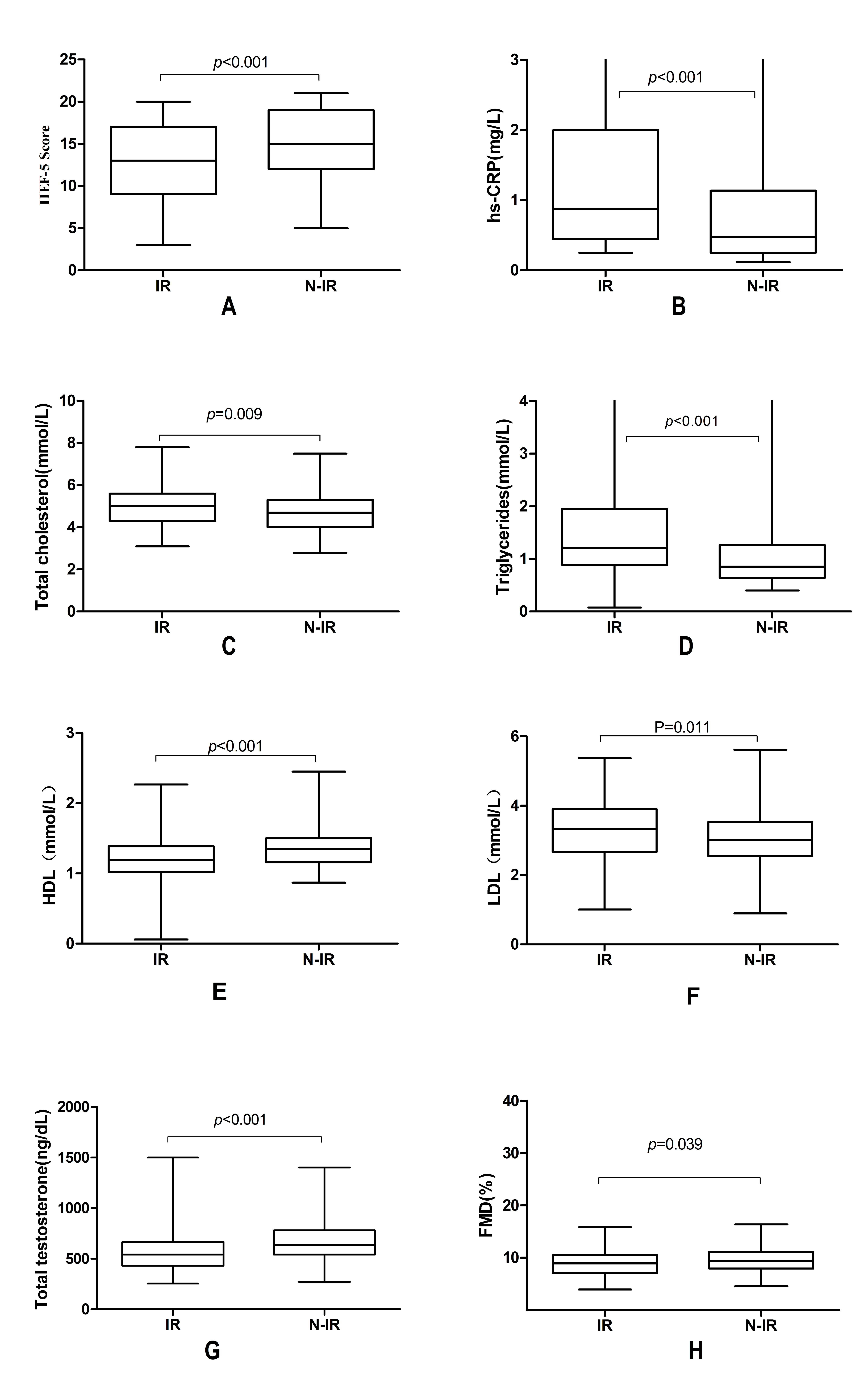

Supplement: Figure S1 — The comparison of IIEF-5 score, lipid profile, hs-CRP, total testosterone, FMD% between IR group and non-IR group. (TIF) [file pone.0083951.s001.tif]
